# Strontium substituted bioactive glasses for tissue engineered scaffolds: the importance of octacalcium phosphate

**DOI:** 10.1007/s10856-015-5653-6

**Published:** 2015-12-24

**Authors:** Danujan Sriranganathan, Nasima Kanwal, Karin A. Hing, Robert G. Hill

**Affiliations:** School of Medicine, Barts and The London School of Medicine and Dentistry, Queen Mary University of London, Turner Street, London, E1 2AD UK; Dental Physical Sciences, Dental Institute, Barts and The London School of Medicine and Dentistry, Queen Mary University of London, Mile End Road, London, E1 4NS UK; School of Engineering and Materials Science, Queen Mary University of London, Mile End Road, London, E1 4NS UK

## Abstract

Porous bioactive glasses are attractive for use as bone scaffolds. There is increasing interest in strontium containing bone grafts, since strontium ions are known to up-regulate osteoblasts and down regulate osteoclasts. This paper investigates the influence of partial to full substitution of strontium for calcium on the dissolution and phase formation of a multicomponent high phosphate content bioactive glass. The glasses were synthesised by a high temperature melt quench route and ground to a powder of <38 microns. The dissolution of this powder and its ability to form apatite like phases after immersion in Tris buffer (pH 7.4) and simulated body fluid (SBF) was followed by inductively coupled plasma optical emission spectroscopy (ICP), Fourier transform infra red spectroscopy (FTIR), X-ray powder diffraction (XRD) and ^31^P solid state nuclear magnetic resonance spectroscopy up to 42 days of immersion. ICP indicated that all three glasses dissolved at approximately the same rate. The all calcium (SP-0Sr-35Ca) glass showed evidence of apatite like phase formation in both Tris buffer and SBF, as demonstrated after 3 days by FTIR and XRD analysis of the precipitate that formed during the acellular dissolution bioactivity studies. The strontium substituted SP-17Sr-17Ca glass showed no clear evidence of apatite like phase formation in Tris, but evidence of an apatite like phase was observed after 7 days incubation in SBF. The SP-35Sr-0Ca glass formed a new crystalline phase termed “X Phase” in Tris buffer which FTIR indicated was a form of crystalline orthophosphate. The SP-35Sr-0Ca glass appeared to support apatite like phase formation in SBF by 28 days incubation. The results indicate that strontium substitution for calcium in high phosphate content bioactive glasses can retard apatite like phase formation. It is proposed that apatite formation with high phosphate bioactive glasses occurs via an octacalcium phosphate (OCP) precursor phase that subsequently transforms to apatite. The equivalent octa-strontium phosphate does not exist and consequently in the absence of calcium, apatite formation does not occur. The amount of strontium that can be substituted for calcium in OCP probably determines the amount of strontium in the final apatite phase and the speed with which it forms.

## Introduction

A bone graft is a material that is surgically implanted into an in vivo environment to aid the regeneration of bone tissue to aid healing of a fracture or bone defect. Bone grafts are designed to be surgically implanted into bone to stimulate osteoregeneration and repair. There are many different types of bone grafts, which are either harvested naturally or produced synthetically [1]. The natural sources of bone graft can be either allograft, xenograft or autograft with the latter being considered the gold standard. However, each of these bone sources has disadvantages; including disease transmission, rejection and limited supply which limits their use [2]. The issues particular to each of these sources has been explored extensively in the literature [1–4]. Producing bone graft materials synthetically is regarded as a more reliable, reproducible, safer method of supplying material for surgery, with the potential to be chemically and structurally optimised to match or surpass the properties of bone grafts from natural sources.

An ideal bone graft will stimulate bone healing, while being capable of undergoing controlled remodelling to ultimately result in the formation of new bone in tandem with the graft disappearing, as required. In addition, the bone graft will initially provide structural stability so that there is not any excessive stress or micromotion in the vicinity of the defect site, which may slow bone healing. The bone graft will also be biocompatible, cheap and easy to use. For optimum bone healing there are three elements which are essential. Osteogenic cells, such as osteoblasts and osteoprogenitor cells in addition to phagocytic cells such as macrophages and osteoclasts are required to ensure that balanced new bone formation and bone remodelling occurs. In addition, growth factors are needed to provide the osteoinductive signals to activate the osteogenic cells [3]. The efficacy of a bone graft is measured by its level of osteoinduction, osteoconduction and osseointegration [4].

Synthetically produced bone graft materials are termed bone graft substitutes with one of the most actively studied among surgeons and researchers being the bioactive glasses. The original bioactive glass, Bioglass^®^ 45S5, was invented in 1969 and still remains the gold standard in bioactive glasses. The compositions of some of the most widely studied bioactive glasses along with the glasses that will be investigated in this paper are shown in Table 1.

**Table 1:** Glass composition (mol%) and network connectivity (NC)

Glass	45S5	13–93	ICIE 16	Stronbone P™ (SP-17Sr-17Ca)	SP-0Sr-35Ca	SP-35Sr-0Ca
SiO_2_	46.13	54.6	49.46	44.5	44.5	44.5
P_2_O_5_	2.6	1.7	1.07	4.5	4.5	4.5
Na_2_O	24.35	6	6.6	4	4	4
K_2_O	0	7.9	6.6	4	4	4
CaO	26.91	22.1	36.27	17.8	35.6	0
SrO	0	0	0	17.8	0	35.6
MgO	0	7.7	0	7.5	7.5	7.5
NC	2.12	2.59	2.13	2.31	2.31	2.31

For bioactive glasses to be effective as bone graft substitutes they must be sintered into porous scaffolds without crystallisation occurring. They essentially act as a temporary template to guide the healing of the defect. They help to stimulate vascularised bone growth and support bone regeneration. The size of the sintering window is important, with a large window allowing porous scaffolds to be produced more easily. In addition to this there are a number of other properties that a scaffold must have to be successful [5].

A bone scaffold needs to be biocompatible in both its original form and in its degradation products so that it does not produce toxic by-products in vivo or activate the immune system of the host, which will result in it being rejected. The scaffold also needs to form a chemical bond to the native bone as this will increase the structural stability of the defect site and is vital to its incorporation into the function structure of the regenerated bone. In addition, the mechanical properties of the scaffold need to be similar to or lower than the native bone to ensure that the stress is spread equally across both the scaffold and the native bone. This is important as if this were not the case then over time bone regeneration may be impaired or reversed. The stability of the structure is also influenced by the rate at which the scaffold degrades which needs to be at the same rate at which new bone is formed, and this is preferably cell mediated through a natural remodelling process. The physico-chemistry of the surface of the scaffold is also important as it needs to facilitate osteogenic cell attachment, as well as stimulating osteoprogenitor cells to produce a bone matrix [6–8].

One of the most important factors that determine the success of a scaffold is its pores. The macropores need to be open and interconnected so that angiogenesis can occur. This is vital as the newly formed bone cannot survive without a viable blood supply. However, if the macropores are too large then the structural integrity of the scaffold will be compromised. A number of studies have shown that an interconnected pore structure is critical to the success of a synthetic bone graft [9]. The macropore interconnections of a scaffold with an osteoconductive chemistry need only to be large enough to allow new bone growth and its associated contiguous vascular network to penetrate freely through the scaffold.

Bioglass^®^ 45S5 forms a carbonate-substituted hydroxyapatite layer on its surface in vivo which forms a strong bond with the native tissues and bones. The new bone that is formed will replace the Bioglass^®^ which degrades in the body over time. The degradation time depends on the way the Bioglass^®^ scaffold was sintered with various models being proposed in the literature to predict this with one example being from a study by Sanz-Herrera and Boccaccini [10]. The degradation will also cause the release of silicon ions as well as other ions such as calcium and sodium [11]. However, the release of ions may not always be a positive characteristic of Bioglass^®^ as in the early stages of degradation there is a very fast release of ions, which may cause changes in pH, which may disrupt the homeostasis of the local environment [12]. Another limitation of Bioglass^®^ is that there are difficulties in producing porous scaffolds from it. The main reason for this is that there is a small difference between its glass transition temperature and its crystallisation temperature, the so called processing or sintering window (defined as the onset temperature for crystallisation minus the glass transition temperature. These difficulties can lead to the formation of a scaffold that has a low strength/cohesivity, [13]. Another limitation is that Bioglass^®^ 45S5 has a slow degradation rate and HA formation rate when compared to other bioactive glasses, which makes it hard to predict the rate of new tissue formation in vivo [14].

The 13–93 composition is based on Bioglass^®^ but differs in that it has a higher SiO_2_ content. Another distinguishing feature of the composition is the low phosphorus content. Studies by O’Donnell, et al. [15] have shown that with increasing phosphorus content there is faster apatite formation and a smaller pH rise. This is advantageous as it maintains the stability of the internal environment and there is an increased bioactivity. Thus, the low PO_4_ content of 13–93 could be considered disadvantageous. Another disadvantage is that the 13–93 composition degrades at a rate that is even slower than Bioglass^®^ 45S5. However, an advantage that 13–93 has over Bioglass^®^ 45S5 is that it has a much larger sintering window which means that porous 3D scaffolds can be created and sintered without crystallisation occurring. This makes it easier to turn 13–93 into scaffolds for implantation in vivo [16]. The large sintering window can be attributed to the presence of magnesium oxide. A study conducted by Watts et al. [17] showed that magnesium oxide extends the sintering window by inhibiting crystallisation but the side effect is that it limits bioactivity by reducing apatite formation. Thus, in formulating the 13–93 glass a compromise was made between its level of bioactivity and its ability to be made into a scaffold.

ICIE 16 is a bioactive glass composition that was designed by Wu et al. [18] primarily not to crystallise during sintering, while having similar bioactivity to the 45S5 composition. This eliminates one of the major disadvantages of Bioglass^®^. Analysis of the ICIE16 composition found that the sintering window was >200 °C, which is more than double that of the 45S5 composition, which is just below 100 °C. It was also found that apatite formation in ICIE16 was accelerated as compared to that of the 13–93 composition, matching the rates observed for 45S5. The explanation proposed for this behaviour was that ICIE16 had a similar network connectivity to 45S5, which is lower than that of 13–93. Network connectivity (NC) is important as higher values result in slower ion exchange and dissolution, which are important steps in the apatite formation mechanism. The NC for the different glass compositions are given in Table 1 [18].

In recent years the positive effects of strontium on bone metabolism have become widely recognised. Strontium ions have been found to inhibit osteoclast activity, while promoting osteoblast activity, which facilitates bone formation. Strontium has also been found to have a mild bactericidal effect at the same concentration that it up regulates osteoblast activity, which is a very advantageous property [19, 20]. However, too much strontium can have negative effects. The inhibition of too many osteoclast cells will inhibit bone regeneration and bone remodelling. The lack of remodelling and regeneration can lead to osteonecrosis and can cause the bones to become brittle. Thus, it is important to ensure an optimal level of strontium in the candidate glass composition [21]. Analysis of strontium ions show that it is very similar, chemically, to calcium, as they are both in group II of the periodic table, resulting in similar electrochemical properties. They are also somewhat similar in size with strontium ions having an ionic radius of 1.16 Å and calcium ions having a radius of 0.94 Å. Due to the similarities with calcium and the desirable properties that strontium possesses, it was hypothesised that strontium oxide (SrO) could be substituted for calcium oxide (CaO) in Bioglass^®^ to combine the positive properties of both Bioglass^®^ and strontium [22].

A series of strontium containing bioactive glasses were patented by Hill and Stevens [23] and one of these compositions was then subsequently turned into a commercial product called Stronbone™ by RepRegen Ltd, UK. Stronbone-P™ is a variant of the original Stronbone™ with the “P” in its name indicating the fact that it is a more porous version. In order to make a porous version and to enable sintering the chemical composition of the glass had to be modified to increase the size of the processing or sintering window to 174 °C, which makes it ideal for forming scaffolds and sintering. Preliminary testing has shown that Stronbone-P™ has a more rapid bone growth and remodelling rate than the original Stronbone™.

In this paper the findings of in vitro studies carried out to assess the ‘bioactivity’ in terms of the acellular dissolution and reprecipitation behaviour of Stronbone-P™ will be reported. The powder version of bioactive glasses were used instead of the sintered scaffold version so that the dissolution and reprecipitation rate of the glass alone could be evaluated. It is a commonly reported finding that forming an scaffold can affect this rate [12]. This is clinically relevant as the powder form can have dental applications such as in toothpaste formulations. Stronbone-P™ was analysed in both simulated body fluid (SBF) and Tris buffer solution for various time points up to 42 days. The decision was also taken to produce two variants of Stronbone-P™, in order to directly assess the effect of the presence of calcium and/or strontium on apatite forming ability. An all strontium version where the calcium is completely replaced with strontium to produce a strontium only Stronbone P™ glass, was called the SP-35Sr0Ca. The second variant had the strontium replaced with calcium to produce a calcium only Stronbone P™, was called the SP-0Sr35Ca. To aid clarity, for the remainder of this paper Stronbone-P™ shall be referred to as the SP-17Sr17Ca to maintain uniformity with the other two variants that are named according to the relative percentage of strontium they contain.

## Materials and methods

The three glass compositions studied are given in Table 1. Proportions of the relevant oxides were mixed together in a platinum crucible and heated to 1460 °C for SP-17Sr17Ca, 1450 °C for SP-0Sr35Ca or 1470 °C for SP-35Sr0Ca. The different temperatures used reflected the effect that strontium has on the melting temperature. Once the furnace was at the desired temperature the glass was held for 1 h and then quenched using deionised water. The coarse granular frit glass was collected, dried overnight and ground into powder using a Gy-Ro Mill (Glen Creston Ltd, Twickenham UK) and sieved using an Endecotts EFL 2000/1 automated sieve shaker to separate the powder into a fine (<38 μm) and coarse frit. The fine and coarse frit were then analysed using differential scanning calorimetry (DSC) to determine the glass transition temperature (Tg) crystallisation onset temperature (Tconset) and processing window (defined as Tconset-Tg).

The acellular ‘bioactivity’ dissolution/reprecipitation tests were conducted at 37 °C in a rollerball incubator with a rotational speed of 60 rpm, using 150 mg fine frit glass incubated for periods of 1, 3, 7, 14, 28 and 42 days in 100 mL of either Tris buffer solution or simulated body fluid (SBF) [24]. After the various time intervals had passed the solution was filtered and refrigerated prior to analysis. Inductively coupled plasma optical emission spectroscopy (ICP-OES) was carried out to monitor ion release at different time points. The solid precipitate formed on the bioglass frit during the bioactivity tests was collected during the solution filtration step along with any remaining frit, dried in an oven at 37 °C for 24 h and together analysed for glass degradation and apatite formation using Fourier transform infrared (FTIR) and X-ray diffraction (XRD) as previously described [25], in addition to ^31^P magic angle spinning nuclear magnetic resonance (MAS-NMR).

^31^P MAS NMR spectra were acquired on a Bruker Avance 600 spectrometer at the Larmor frequency of 242.96 MHz. All spectra were acquired with a single 90^o^ pulse. Samples were packed into 4 mm outer diameter Zirconia rotors and spun at the magic angle at a spinning speed of 12 kHz. Each spectrum is a sum of 16 scans acquired with a 60 s recycle delay. Spectra were externally referenced to 85 % phosphoric solution at 0 ppm.

## Results and discussion

**Fig. 1:**
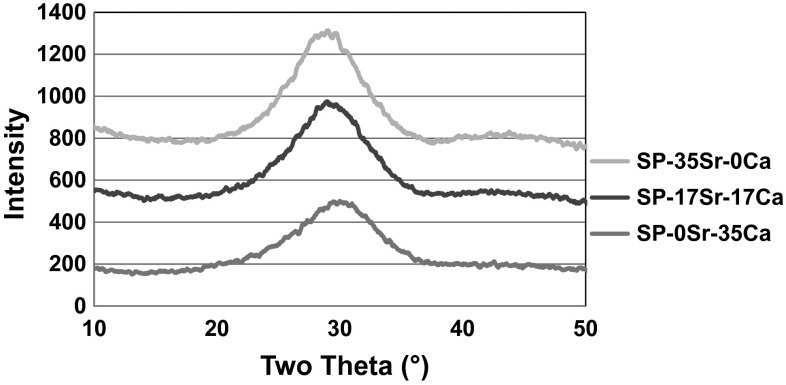
XRD of coarse frit glasses

The XRD trace in Fig. 1 shows that all the glass compositions under investigation were amorphous, with the presence of a broad amorphous peak as is typical of bioactive glasses. It was also evident that the amorphous peak had a higher intensity and was shifted more to lower 2θ values with increasing strontium content. The intensity variation could be attributed to the fact that strontium has a higher atomic number than calcium and thus scatters X-rays more effectively. While the shift towards lower 2θ values could be explained by considering the Braggs diffraction equation. The equation is ηγ = 2dsinθ with “ηγ” being a fixed value and “d” being the space between the atoms of the glass. As strontium is a larger atom than calcium, the spacings in the glass are larger and thus the sinθ value is smaller, which shifts the maximum in the amorphous scattering to lower 2θ values [26, 27].

**Fig. 2:**
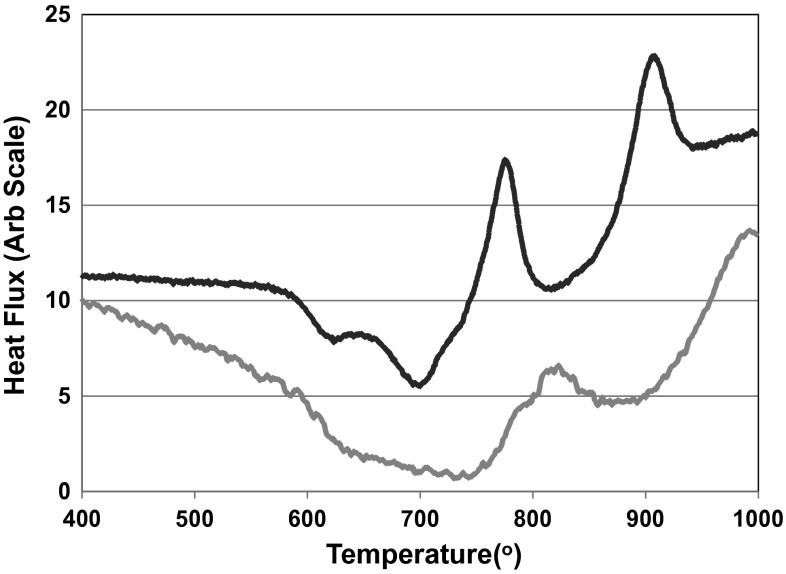
DSC trace of fine powder (*uppermost line*) and coarse frit (*lowermost line*) of the SP-17Sr17Ca

The fine powder and coarse frit have very similar glass transition temperatures (Tg) and large sintering windows as is evident in Fig. 2. The processing windows is larger for the coarse frit which implies that crystallization occurs by surface nucleation, as the coarser frit glass will have lower surface area to volume ratio than the fine frit glass. The large reduction in heat flux following the Tg with fine powder which is absent with coarse frit is a result of the sintering and compaction of the glass and is indicative of the ease of viscous flow sintering that occurs with these glasses.

The results of the DSC analysis show that Tg decreases with increasing strontium content, which is evident in Table 2 and can be explained by the fact that the strontium ion is larger than the calcium ion and results in a less dense glass network [26]. The size of the sintering window is also affected by the presence of strontium with a high level of strontium increasing the sintering window.

**Table 2:** Glass transition and crystallisation onset temperatures for the glasses

Glass	Tg (°C)	Tg−T_cons_ (°C)	T_c_ (°C)
SP-35Sr-0Ca	578	260	874
SP-17Sr-17Ca	614	193	879
SP-0Sr-35Ca	618	247	907

**Fig. 3:**
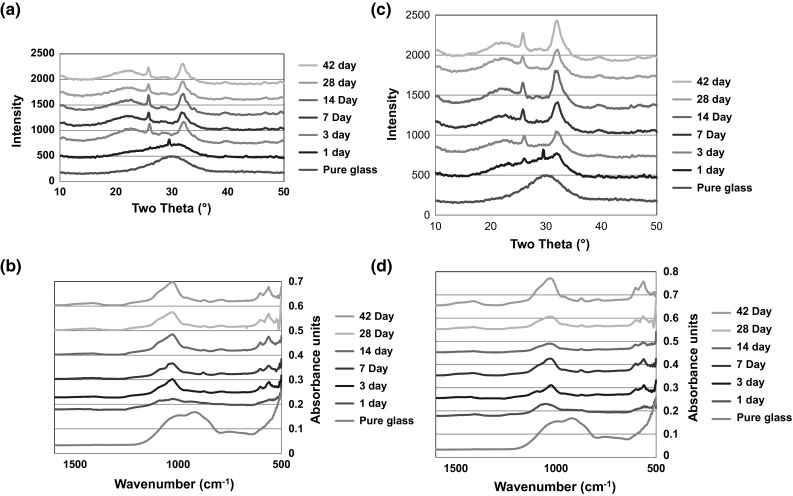
**a** XRD of SP-0Sr35Ca in Tris buffer solution, **b** FTIR of SP-0Sr35Ca in Tris buffer solution, **c** XRD of SP-0Sr35Ca in SBF and **d** FTIR of SP-0Sr35Ca in SBF

There is clear evidence of the formation of an apatite like phase with the SP-0Sr35Ca in Tris buffer solution and SBF when analysed with FTIR and XRD. Figure 3a, c show the appearance of peaks at 26° and 32° from 3 days, which is indicative of apatite formation. This is supported by the split bands at 560, and 600 cm^−1^ from 3 days in the FTIR spectra Fig. 3b and split bands at 570, 612 and 1420 cm^−1^ which are also indicative of the presence of an apatite like phase. In Fig. 3b, d, the changes in the FTIR traces over time shows the loss of non-bridging oxygen and the development of a phosphate peak behaviour that is expected of the glass. The development of the phosphate band also supports the presence of the apatite like phase.

**Fig. 4:**
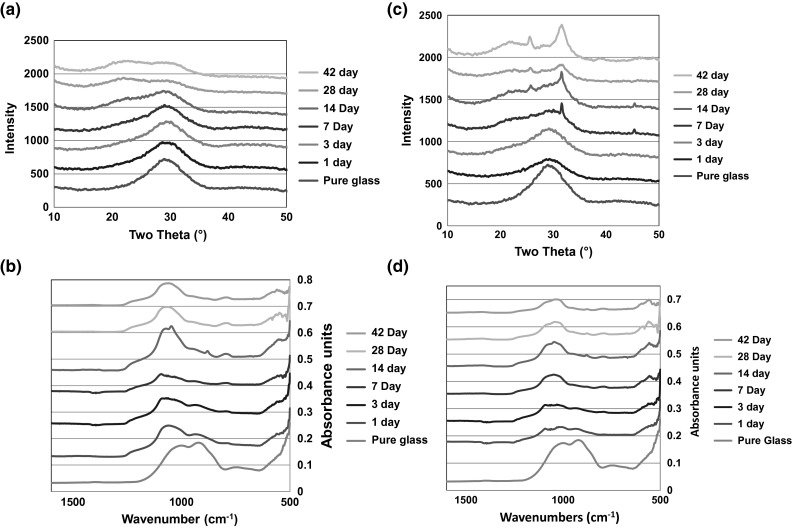
**a** XRD of SP-17Sr17Ca in Tris buffer solution, **b** FTIR of SP-17Sr17Ca in Tris buffer solution, **c** XRD of SP-17Sr17Ca in SBF and **d** FTIR of SP-17Sr17Ca in SBF

The SP-17Sr17Ca shows a marked difference in the XRD results after immersion in SBF and Tris. On incubation in SBF (Fig. 4c) there are clear signs of development of an apatite like phase as two peaks appear at 26° and 32° from 14 days. However, on incubation of the Tris buffer (Fig. 4a) there seems to be the growth of a broad peak centred around 32° which corresponds to three of the principle diffraction lines of apatite but it isn’t very clear. The lack of evidence of the presence of an apatite like phase can be attributed to the lack of calcium in Tris that could be preventing or delaying apatite formation. The FTIR results supports the findings of the XRD with there being signs of apatite formation on incubation in the SBF (Fig. 4d) as split bands are present around 560 and 600 cm^−1^ from 7 days. However, after incubation in the Tris (Fig. 4b) there are only signs of the formation of the split bands from 28 days and the splitting is not very obvious. This is consistent with the XRD results where the diffraction lines for apatite are not very clear.

**Fig. 5:**
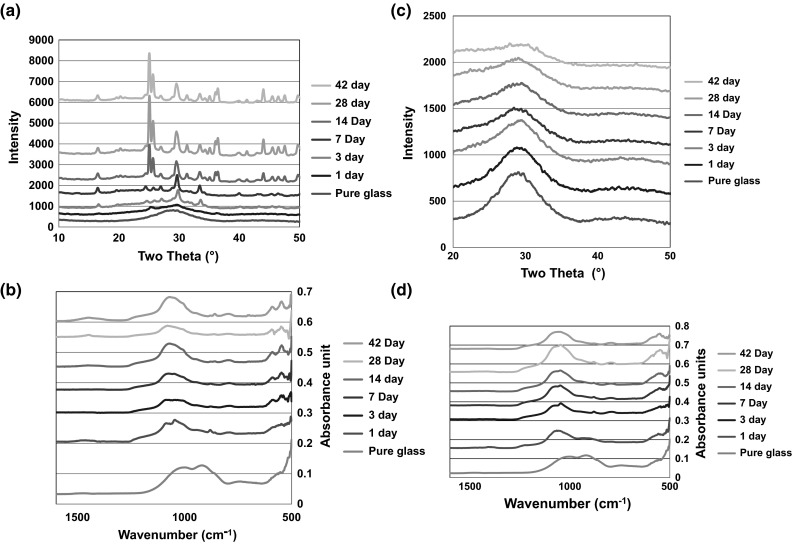
**a** XRD of SP-35Sr0Ca in Tris buffer solution, **b** FTIR of SP-35Sr0Ca in Tris buffer solution, **c** XRD of SP-35Sr0Ca in SBF and **d** FTIR of SP-35Sr0Ca in SBF

The SP-35Sr0Ca behaved differently to both the 0 % and SP-17Sr17Caes. Analysis by XRD (Fig. 5a) of SP-35Sr0Ca exposed to Tris buffer solution demonstrated the appearance of a new highly crystalline phase that cannot be matched to common strontium and/or phosphate based compounds. This new phase appeared after 7 days and is termed “X phase”. X phase did not match Collin’s salt [Sr_6_H_3_(PO_4)5_·2H_2_O], tristrontium phosphate or strontium apatite. It is hypothesised that it is most likely to be some form of strontium orthophosphate as it shares diffraction lines with Collin’s salt [28]. This hypothesis is supported by the FTIR (Fig. 5b) which exhibits split bands at 555, 597 and a band at 1100 cm^−1^ which indicate that there is some type of orthophosphate present [29]. The analysis of the SP-35Sr0Ca in SBF shows no clear signs of apatite formation. In the XRD (Fig. 5c) there is a growth in the region of 30°–33°, at all time points, which is where an apatite phase would be found. FTIR analysis demonstrated the growth of a broad peak at around 1100 cm^−1^ and split bands around 560 and 600 cm^−1^, which could be evidence of the early evolution of an apatite like phase, but it is not very clear.

**Fig. 6:**
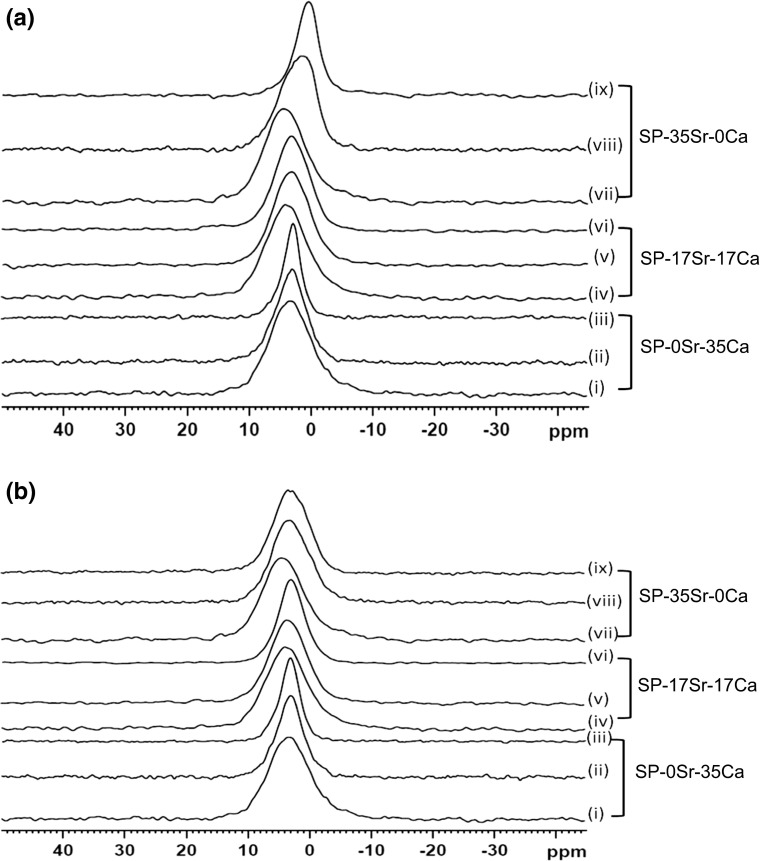
**a** ^31^P NMR spectra of unimmersed coarse powder, fine frit after 3 days immersion and fine frit after 28 days immersion for SP-0Sr35Ca (*i*, *ii*, *iii*), SP-17Sr17Ca (*iv*, *v*, *vi*) and SP-35Sr0Ca (*vii*, *viii*, *ix*) in Tris buffer solution. **b** ^31^P NMR spectra of unimmersed coarse powder, fine frit after 3 days immersion and fine frit after 28 days immersion for SP-0Sr35Ca (*i*, *ii*, *iii*), SP-17Sr17Ca (*iv*, *v*, *vi*) and SP-35Sr0Ca (*vii*, *viii*, *ix*) in simulated body fluid

The spectra remain fairly broad even after immersion for 28 days both in Tris buffer and SBF, except for SP-0Sr35Ca after 28 days, however there is a shift in peak position compared to untreated glass. Chemical shift goes down to about 3 ppm which is an indication of apatite like phase formation [30]. Analysis of SP-0Sr35Ca after 28 days immersion (Fig. 6a, b, iii) shows a much sharper peak with chemical shift of 2.8 ppm which is a clear indication of apatite formation. In agreement with XRD and FTIR results, the SP-35Sr0Ca shows unexpected results with a very low chemical shift of 0.5 ppm in Tris buffer (Fig. 6b, iv–vi) which is an indication of some acidic orthophosphate formation, however in SBF (Fig. 6b, iv–vi) spectral peaks are broad and fairly symmetric with chemical shift centred at about 3 ppm.

**Fig. 7:**
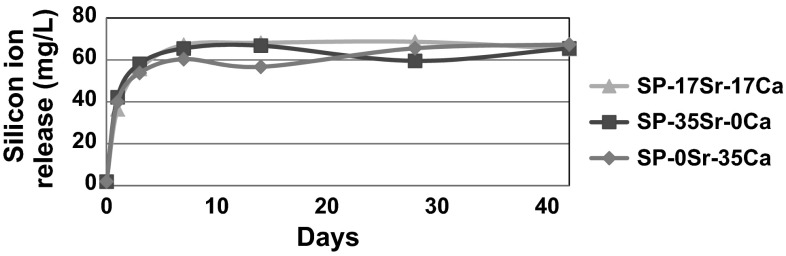
Silicon ion release in Tris buffer solution for the SP-0Sr-35Ca, SP-17Sr-17Ca and SP-35Sr-0Ca formulations over the 42 day period

The ICP analysis is consistent with the XRD and FTIR with phosphorus and calcium levels reducing as expected when an apatite like phase was being produced. The silicon release is very similar for all the glasses in both immersion media. This can be clearly seen in Fig. 7 where there is an increase in silicon as the glasses dissolve and then there is a plateau at around 60 mg/L. In all of the glasses, in the first 5 days there is a rapid release of silicon which then slows to reach a plateau at 7 days. This level of silicon release is not concerning as there are no reported symptoms or toxic effects on the human body in the literature and although its exact role in the body has not been fully ascertained, it does play a role in collagen synthesis and bone matrix development [31]. Another assumption that can be made is that as the dissolution rate of the glasses are not significantly different, the apatite like phase formation must be attributed to the glass compositions themselves and not as a result of the glasses dissolving at different rates. This is supported by all the XRD data where there is a consistent growth in all the glasses at around 23° 2θ, which signifies the growth of an amorphous peak that is thought to correspond to silica gel. This occurs simultaneously with the loss of scattering around 28° as the glass dissolves. Another feature of the XRD data that is consistent is that glasses immersed in SBF have broader peaks than the glasses immersed in Tris. This can be attributed to the presence of magnesium ions in the SBF that reduces the apatite crystallite size and causes Scherrerr line broadening. Scherrerr line broadening also explains why the peaks sharpen at the later time points in the XRD data, corresponding to crystallite growth time [32].

Octacalcium phosphate, Ca_8_(PO_4_)_6_H_2_·5H_2_O (OCP) is thought to be a precursor to apatite formation with evidence of OCP formation found in the natural mineralisation processes of enamel and bone [33–36]. Whilst hydroxyapatite is the thermodynamically most stable phase, OCP is thought to form because it has a lower activation energy of nucleation, as a result of the water layer in its structure reducing the interfacial surface energy upon nucleation in water. OCP has a crystal structure very similar to hydroxyapatite, and X-ray diffraction patterns for OCP and hydroxyapatite are almost indistinguishable [34]. The only major difference being a diffraction peak at 4.6° 2θ (with CuKα X-rays), corresponding to the water layer which is in OCP, but not HA. This diffraction peak is problematic to detect since it is (i) at a very small angle, (ii) tends to broaden out as a result of any structural disorder and (iii) OCP tends to form as very thin plates in this crystallographic direction resulting in chronic Scherrer line broadening [33–36]. To make detection of OCP even more problematic OCP is not thermodynamically stable and drying it or putting it under vacuum favours the loss of water and conversion to an apatite. It is worth considering that OCP and hydroxyapatite are two extreme members in a spectrum of calcium phosphates whose exact chemical composition probably depends on the pH conditions and degree of supersaturation (33, 35). The formation of OCP is generally favoured by less basic pHs and higher P:Ca ratios [35].

OCP is thought to form in the SP-0Sr35Ca and the SP-17Sr17Ca. It appears as if a small amount of strontium can be substituted into the OCP crystal lattice without destabilising it. However, large amounts of strontium are unlikely to be substituted into the lattice as there is not a recognised stable form of octa strontium phosphate. At above a certain degree of strontium substitution for calcium in OCP, the OCP becomes unstable. This extreme scenario occurs with the SP-35Sr0Ca in Tris buffer, since there is no source of calcium, OCP cannot form and instead a new crystalline strontium orthophosphate forms. While in SBF apatite formation is inhibited in the presence of the SP-35Sr0Ca, supporting the theory that OCP is a precursor to apatite formation, as calcium is only available from the SBF and the OCP structure is unable to accommodate too great a level of strontium into its lattice without becoming destabilised. This is reflected in results of this paper where the most clear evidence of apatite like phase formation is seen in with the SP-0Sr35Ca, where there are no strontium ions. The apatite like phase formation is less obvious with the SP-17Sr17Ca and it appears as if there is no apatite formation with the SP-35Sr0Ca in Tris. Previous studies conducted by this research group have shown that with increasing strontium concentration the rate of apatite formation increases. A possible explanation for these apparently contradictory results could be that the previous studies were all performed with glasses containing fluoride or low levels of phosphate. Fluoride is thought to eliminate the requirement for an OCP precursor phase during apatite formation [37]. Thus, it might be the case that strontium will only have a positive effect on apatite formation in high phosphate content bioactive glasses when fluorine is also present. However, further studies with fluorine substituted glasses of the compositions used in this paper are required in order to test this hypothesis. A previous study by Gentleman et al. [38], investigating strontium incorporation into bioactive glasses used low phosphate contents of 1.07 and 2.6 mol% P_2_O_5_ compared to the higher level of 4.5 mol% used in the glasses of the present study. The low phosphate content coupled with the much higher pH rise with these glasses and the fact these studies were carried out in SBF is likely to favour the direct formation of a hydroxycarbonated like apatite, rather than going via a precursor OCP route. At high pH > 9 direct formation of a hydroxyapatite is thought to occur and under these conditions strontium exhibits complete solid substitution for calcium in the hydroxyapatite lattice [26].

## Conclusion

The results indicate that increasing the substitution of calcium for strontium in high phosphate bioactive glasses appears to retard the formation of an apatite like phase. It is proposed that apatite formation proceeds via the formation of an octacalcium phosphate precursor phase, which then transforms towards a hydroxycarbonated apatite. Above a certain concentration of Sr or Sr/Ca ratio, the Octacalcium phosphate precursor phase is unable to form which ultimately retards the formation of a hydroxycarbonate like phase. The complete absence of calcium in the SP-35Sr-0Ca glass in Tris buffer results in a completely new orthophosphate phase, which could not be identified. Further experiments are required to determine the structure of this new phase as it may provide further clues into understanding the role of Sr on bone formation. Identifying the optimum level of strontium substitution is also important as it will allow the formulation of Stronbone P™ to be improved.
